# Remarks on the Unity of the Body, as Illustrated by Some of the More Striking Phænomena of Sympathy, Both Mental and Corporeal, with a View of Enlarging the Grounds and Improving the Application of the Constitutional Treatment of Local Diseases

**Published:** 1836-07

**Authors:** 


Art. III.—*Remarks on the Unity of the Body, as illustrated by some of the more striking Phænomena of Sympathy, both Mental and Corporeal, with a view of enlarging the Grounds and improving the Application of the Constitutional Treatment of Local Diseases.* By George Macilwain, Surgeon to the Finsbury Dispensary.—*London,* 1836. 8vo. pp. 294.

The title of this book is a fair epitome of its contents. Mr. Macilwain's object has been to illustrate and enforce the importance of the principle which seemed to John Hunter and Abernethy of so much value—that the whole of the body sympathizes with all its parts. In discussing sympathies, Mr. M. has followed generally John Hunter's plan of describing the more prominent ones without attempting to pursue the enquiry so ably commenced by Whytt, and to which modern physiologists have attached considerable importance,—the influence of the brain and spinal cord in their production. Mr. M. has framed a classification of sympathies rather as a convenient help to memory than as an attempt at a scientific arrangement, which at present he thinks impossible. The first division consists of *Structural Sympathies,* or of parts possessing a similar structure; including "sympathies of mechanical relation," as disordered action of the heart producing hepatic congestion; the second division is founded on indentity or analogy of function, or *Functional Sympathies,*

as between the uterus and mammary glands; the third division is that of *Intermediate Sympathies,* as when the stomach sympathizes with the uterus through the medium of the nervous system; a fourth includes *Anomalous Sympathies,* so often found connected with the digestive organs, and which will not fall under the other heads, such as potatoes in one individual invariably producing a paroxysm of pain in the bladder. The sympathies of the brain, including the mental sympathies, are separately considered. Each of these general divisions is illustrated by examples. This arrangement wholly fails in the simple object it professes, of assisting the memory; as it obscures the whole subject by breaking it up into so many parts that repetition is unavoidable. Thus, to instance sympathies of the mucous membrane of the alimentary canal under the structural division, allusion is made to the sympathy of the stomach with other portions of the canal, and of these with the bronchial mucous membrane, and that lining the nose, with the skin, &c.; of the urethra and conjunctiva in gonorrhœa and others: in the functional division all this is repeated; and under the head "Intermediate Sympathy," that sympathy between the stomach and lungs which gives rise to "stomach cough" is placed, from a supposition that it is brought about by the intermediate agency of the skin. Had Mr. Macilwain contented himself with fully describing under one division the sympathies of each organ, as he has done that of the brain, he would have avoided this needless repetition, and would have been in the same proportion clearer. The separation of a few sympathies under the title of "Intermediate" is particularly objectionable, as it is far from improbable that all sympathies are intermediate, that they all take place through the medium of the brain and spinal marrow. Even if this exclusive view is not adopted, yet still a much larger number of sympathies than are classed under this head can be explained most satisfactorily by the above theory.

The illustrations of Mr. Macilwain are not intended to include all the sympathies, but merely the more prominent ones. They bear the marks of considerable reflection, and attention to the subject. General statements however are too often substituted for particular facts, which are so much more calculated to make an impression.

From the consideration of the different sympathies Mr. Macilwain passes to their application to the elucidation of the causes of disease. The larger number of diseases (he says,) are produced by the application of cold and moisture: the normal effect of this is reaction; but, when this fails to take place, either from the state of the individual or the long continued application of the agents, there is a sense of chilliness: if this is allowed to continue, the reaction which ordinarily takes place in the skin is succeeded by one analogous in some other part, and generally in some one with which the skin sympathizes, or which has been previously diseased, or weakened.

"Now, that this is sympathy, the remedies which are successful, as well as the history of disease, alike demonstrate. Say that the primary affection of the skin produces an affection of the throat; what is it but an increased vascular action, *analogous* to that which should have taken place on the skin? The patient applies a warm stocking to his throat, gets into a warm bed, and often rises in the morning nearly or quite well." * * * * *

"Should the part which sympathizes with the skin be previously disordered, the sympathetic excitement is much more serious; if the part thus sympathetically attacked be previously diseased, or should the general health have been previously disturbed, the consequences are frequently fatal. Persons having a disposition to bronchitis, illustrate the former position; chronic disease of the larynx, or tuberculated lung, the latter." (P. 131.)

It frequently happens that different results take place in the same individual from the same cause. Mr. Macilwain thinks that this is to be explained by the different conditions of the nervous system, "because we can conceive no other source through which external influences make any impression at all:" and he further supposes that these differences depend greatly on two causes almost peculiar to man in his civilized condition—depressing and exciting moral causes, and disorders of the stomach; the former being in direct connexion with the nervous system, and the latter being intimately related to it. Whilst other organs which may originate disease "are in a *comparatively* natural state, as to external influences, having seldom to counteract any to which they are not necessarily or naturally exposed; the digestive organs, on the contrary, are subject to every possible variety of adaptation of function, from factitious causes of derangement; so numerous, and so varied, as to be alike but in one feature, that they are generally contrary to nature and to common sense." (P. 148.)

"Not content with satisfying the suggestions of nature, by one kind of food, every meal often becomes the occasion of factitious stimulation. Sometimes by variety of condiments; sometimes by direct stimuli. Does excitement produce weakness, the jaded organ is stimulated anew. If the ingenuity of the epicure, or the power of the gourmand, fail, medicine too often lends its aid as a soi-disant votary of science to the general work of excitement; and tonics, cordials, dinner pills, and a variety of ministerings to the fatigued sensualist, lull those healthy cautions which the sensations of nature suggest, and sustain for a little a transitory enjoyment, only more surely to complete a functional derangement which luxury had begun." (P. 147.)

The particular application of these views of sympathy to treatment, which Mr. Macilwain especially dwells on, is, that in numerous instances in attempting to cure disease we can effect our aim by directing our attention and remedies to the organs which sympathize with the one primarily diseased, particularly when more direct measures have failed or are unadvisable. Details of treatment in accordance with this principle are not given, but, from a few cases of patients with various nervous symptoms, and disorder of the general health, we find that Mr. Macilwain's practice is to ensure regularity of the functions of the chylopoietic viscera, to diminish their labour, and to avoid disturbances by great attention to diet, warm water clysters, and such simple means; and acting on the skin by gentle diaphoretics, warm clothing and vapour baths. In one case of extensively ulcerated leg, the urine was generally thick, although the appetite was good, bowels regular, &c. "I now said to the pupil (for this was a patient of the Dispensary,) just act a little on her kidney, and see the effect on the leg; but let her omit all her other medicine, and only take a diuretic." The whole ulcer was cicatrized in a week. In another case where strumous ophthalmia was attended with extreme intolerance of light, which had resisted the usual constitutional treatment and counter-irritants, this symptom yielded to a plaster with tartarized antimony applied to the epigastrium.

This kind of treatment, which Mr. M. calls "working by the sympathies," has, we thoroughly believe, the high recommendation of being the usual practice of all judicious practitioners, and we here probably differ from Mr. Macilwain, who evidently believes he has more originality than we can give him credit for. As far as we can decide from our own observation of the general practice of well-educated men, the importance of attending to constitutional treatment in all diseases seems to be duly recognized, although all may not carry their views of sympathy to so great an extent as Mr. Macilwain, or even be aware they are working by the sympathies at all when using the same means. Mr. Macilwain attaches an undue importance to his theoretical explanations of disease; cautious theories which explain satisfactorily, according to the actual state of pathology, any series of phenomena, are not to be undervalued, but they should be held loosely. The little relative importance of such explanations, when compared with the faithful observations of facts, is strongly exemplified in the writings of Sydenham. His histories of disease, and the treatment recommended, will continue to be valuable as long as medicine is studied; whilst the theories of the causes of the same diseases, and the explanations of the effects of the remedies, the product of the same man, are now worthless.

Mr. Macilwain has not the pen of a ready writer: his style is turgid and parenthetical, the sentences are occasionally of extraordinary length, and the words often appear to be selected for the same reason; his arguments, too, are diffuse and involved. On these accounts, the results of much reflection fail to make their due impression, and "plain things are inflated into marvels." The leading principle which he has endeavoured to inculcate, the unity of the body, is usefully enforced at the present time, when the local changes of disease are perhaps studied with too exclusive an attention.

An introductory lecture to a course of surgery concludes the volume: it contains much useful advice to students, and a just tribute of praise to Abernethy.

